# A42 SUCCESSFUL ENDOSCOPIC ASSISTED LYMPHATIC EMBOLIZATION IN THE MANAGEMENT OF FONTAN REPAIR ASSOCIATED PROTEIN-LOSING ENTEROPATHY

**DOI:** 10.1093/jcag/gwae059.042

**Published:** 2025-02-10

**Authors:** P Shelemey, J Cooper, J Windram, D McNally, F Hoentjen

**Affiliations:** Gastroenterology, University of Alberta Faculty of Medicine & Dentistry, Edmonton, AB, Canada; Gastroenterology, University of Alberta Faculty of Medicine & Dentistry, Edmonton, AB, Canada; University of Alberta Faculty of Medicine & Dentistry, Edmonton, AB, Canada; University of Alberta Faculty of Medicine & Dentistry, Edmonton, AB, Canada; Gastroenterology, University of Alberta Faculty of Medicine & Dentistry, Edmonton, AB, Canada

## Abstract

**Background:**

Protein-losing enteropathy (PLE) is a known complication in patients who have undergone a Fontan procedure for single ventricle physiology and confers a poor prognosis. PLE is characterized by low levels of albumin, gamma globulins, and other proteins involved in the coagulation cascade. This can result in malnutrition, functional immunodeficiency and increased thrombosis risk. Medical therapy is often challenging in such cases and in refractory cases heart transplantation has traditionally been indicated. Recently however intervention upon abnormalities of the lymphatic system has been proven to be successful in resolving PLE in this patient population.

**Aims:**

We present the case of a 25-year-old patient with a Fontan repair who developed PLE and was treated successfully with endoscopic assisted lymphatic embolization.

**Methods:**

Case Report.

**Results:**

A 25-year-old patient who had undergone a Fontan repair presented with septic arthritis of the right knee. She was noted have features of severe PLE with a significantly low albumin, malnutrition and functionally impairing peripheral edema (Table 1). She was investigated to determine whether lymphatic intervention was a possibility. A magnetic resonance imaging (MRI) lymphangiogram as well as interventional radiological imaging of the lymphatic system confirmed aberrant lymphatic channels as the source of significant lymphatic leakage into the small intestine.

A combined procedure between gastroenterology and interventional radiology (IR) was performed. The patient underwent a push enteroscopy while simultaneous percutaneous intrahepatic lymphatic access was obtained by IR. Methylene blue was injected into the lymphatic system and leaked through the abberant hepatoduodenal lymphatic channel into the intestinal lumen. The sites of leakage were viewed endoscopically at the duodenal sweep and cap. The scope tip was positioned abutting each leaking site to allow localization by IR. These sites were embolized with glue until no more methylene blue was seen leaking into the lumen.

The patient made significant improvements in the weeks leading up to discharge (Table 1). Today, she is well, maintaining a stable weight and her malnutrition and edema have resolved.

**Conclusions:**

This case highlights the need for multi-disciplinary care in such complex patients. The presentation of PLE in patients with a Fontan repair can now be successfully intervened upon through lymphatic intervention. Direct endoscopic visualization to localize sites of lymphatic leakage in cases of PLE is essential to allow for targeted IR embolization. This case aims to bring awareness of this technique to the wider gastroenterology community.

Table 1. Biochemical and objective markers associated with malnutrition and PLE on Admission, Discharge, and at Follow Up (four months post discharge). Retention of serum proteins improved and excretion of alpha 1 antitrypsin decreased. Weight improved as the patient’s peripheral edema resolved.

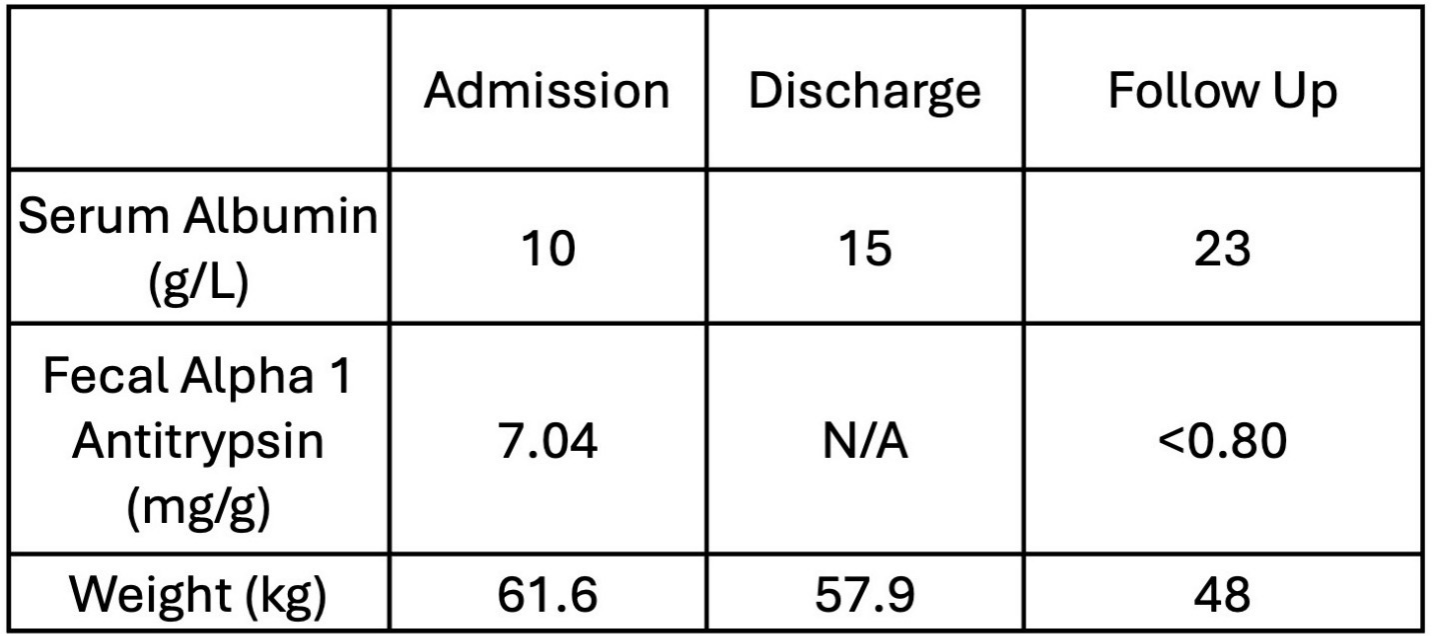

**Funding Agencies:**

